# Schisandrol A Attenuates Myocardial Ischemia/Reperfusion-Induced Myocardial Apoptosis through Upregulation of 14-3-3*θ*

**DOI:** 10.1155/2021/5541753

**Published:** 2021-06-26

**Authors:** Shuaishuai Gong, Jincheng Liu, Shiyao Wan, Weiwei Yang, Yuanyuan Zhang, Boyang Yu, Fang Li, Junping Kou

**Affiliations:** Jiangsu Key Laboratory of TCM Evaluation and Translational Research, Research Center for Traceability and Standardization of TCMs, School of Traditional Chinese Pharmacy, China Pharmaceutical University, Nanjing, China

## Abstract

Schisandrol A (SA), one of the most abundant bioactive lignans extracted from the *Schisandra chinensis* (Turcz.) Baill., has multiple pharmacological properties. However, the underlying mechanisms of SA in protection against myocardial ischemia/reperfusion (MI/R) injury remain obscure. The present experiment was performed to explore the cardioprotective effects of SA in MI/R injury and hypoxia/reoxygenation- (H/R-) induced cardiomyocyte injury and clarify the potential underlying mechanisms. SA treatment significantly improved MI/R injury as reflected by reduced myocardium infarct size, attenuated histological features, and ameliorated biochemical indicators. In the meantime, SA could profoundly ameliorate oxidative stress damage as evidenced by the higher glutathione peroxidase (GSH-Px) as well as lower malondialdehyde (MDA) and reactive oxygen species (ROS). Additionally, SA alleviated myocardial apoptosis as evidenced by a striking reduction of cleaved caspase-3 expression and increase of Bcl-2/Bax ratio. Further experiments demonstrated that SA had certain binding capability to the key functional protein 14-3-3*θ*. Mechanistically, SA prevented myocardial apoptosis through upregulating 14-3-3*θ* expression. Interestingly, siRNA against 14-3-3*θ* could promote apoptosis of cardiomyocytes, and H/R injury after knockdown of 14-3-3*θ* could further aggravate apoptosis, while overexpression of 14-3-3*θ* could significantly reduce apoptosis induced by H/R injury. Further, 14-3-3*θ* siRNA markedly weakened the antiapoptotic role of SA. Our results demonstrated that SA could exert apparent cardioprotection against MI/R injury and H/R injury, and potential mechanisms might be associated with inhibition of cardiomyocyte apoptosis at least partially through upregulation of 14-3-3*θ*.

## 1. Introduction

Myocardial ischemia/reperfusion (MI/R), a leading cause of death and disability, is caused by the blood recovery after a vital period of coronary artery occlusion, which easily leads to myocardial infarction or heart failure [1]. It is well known that the pathogenesis of MI/R injury is closely associated with oxygen free radical damage, calcium overload, energy deficit, and inflammatory reaction, ultimately leading to cell apoptosis [2, 3]. Thus, inhibition of myocardial apoptosis is a potential therapeutic strategy for MI/R injury.

Schisandrol A (SA) is a bioactive compound with prominent antioxidant activity isolated from *Schisandra chinensis* (Turcz.) Baill. Modern pharmacological studies have shown that SA could protect intestinal epithelial cells from the effects of cytotoxicity, oxidative damage, and inflammatory response induced by deoxynivalenol [4]. Studies have also found that SA could improve H_2_O_2_-induced PC12 cell damage by modulating the NF-*κ*B signaling pathway [5]. Meanwhile, SA could reduce cerebral ischemia/reperfusion injury by suppressing inflammation and oxidative stress [6]. Additionally, SA inhibits autophagy through regulating the AMPK-mTOR signaling pathway and also has a certain protective effect on OGD/R-induced neuronal damage [7]. Moreover, our previous study has found that SA exerted a protective role against acute myocardial ischemia injury and elucidated its potential mechanism from the perspective of metabolomics [8]. However, the protective effect of SA on MI/R injury remains unclear, and further studies are still needed to identify its possible target and elucidate the underlying mechanism.

It is generally accepted that 14-3-3 proteins exist in all eukaryotes and consist of 7 isoforms (*β*, *γ*, *ζ*, *η*, *θ*, *σ*, and *ε*) with unique expression patterns in different cell types and tissues. Accumulating evidence has demonstrated that 14-3-3s are involved in the regulation of diverse physiological and pathological processes including cell cycle, signal transduction, metabolism, protein transport, and apoptosis [9–11]. Correlative studies suggested that anoxic preconditioning could increase 14-3-3 protein expression in neonatal rat cardiomyocytes via extracellular signal-regulated protein kinase 1/2 [12]. Meanwhile, the 14-3-3 isoforms were upregulated in burns and lipopolysaccharide-induced acute rat myocardial injuries [13]. Previous studies have validated that 14-3-3*γ* protein improves lipopolysaccharide-induced myocardial injury through the Bcl-2 family/mitochondria pathway [14]. And capsaicin could prevent mitochondrial damage and protect cardiomyocytes from anoxia/reoxygenation injury via 14-3-3*η*/Bcl-2 [15]. Additionally, prostacyclin protects vascular smooth muscle cell against apoptosis and phenotypic switch via PPAR*α* by upregulating 14-3-3*β* and 14-3-3*θ* [16]. Moreover, it was found that maternal hypoxia increases apoptosis in the fetal rat heart, and 14-3-3*θ* isoform as a compensation was upregulated in chronic hypoxia during pregnancy [17], whereas the effect of 14-3-3*θ* protein on MI/R injury is not clear.

Here, SA was systematically investigated *in vivo* in a MI/R injured mouse model and *in vitro* in H9c2 cardiomyocyte cell line subjected to hypoxia/reoxygenation (H/R) injury, elucidating the cardioprotective role of SA and additionally investigating the potential mechanisms of SA against myocardial apoptosis, especially focusing on whether 14-3-3*θ* might be a potential therapeutic target for the myocardial protection of SA.

## 2. Materials and Methods

### 2.1. Animal Model

Male ICR mice (23-26 g) were obtained from Model Animal Research Centre of Yangzhou University (Yangzhou, Jiangsu, China). The mice were maintained with a standard vivarium with free access to food and water. All animal experiments were approved by the National Institutes of Health *Guide for the Care and Use of Laboratory Animals*, and the protocols used were also consistent with the Animal Ethics Committee of China Pharmaceutical University, Nanjing, China.

Myocardial ischemia/reperfusion injury model was produced as the paper described [18]. In brief, the left anterior descending coronary artery (LAD) was temporarily occluded for 30 min and then released for 24 h. The mice were anesthetized with sodium pentobarbital (50 mg/kg; ip), and the LAD was ligated using a slipknot (6-0 silk). Successful MI/R model was confirmed by the elevation of ST segment on an electrocardiogram monitor. Mice were randomly divided into six groups: (1) the sham group (without LAD ligation, normal saline, ip), (2) the MI/R group (LAD, normal saline, ip), (3) the SA-6 mg/kg group (LAD, SA-6 mg/kg, ip), (4) the SA-12 mg/kg group (LAD, SA-12 mg/kg, ip), (5) the SA-24 mg/kg group (LAD, SA-24 mg/kg, ip), and (6) the metoprolol (Met)-5.14 mg/kg group (LAD, Met-5.14 mg/kg, ig). All drugs were administrated within 10 min prior to reperfusion. And the sham and the MI/R mice were given the same volume of normal saline.

### 2.2. TTC Staining

After reperfusion for 24 h, the heart samples from each group were quickly removed and frozen at -70°C. Then, the tissues were sliced transversely into five slices, which were incubated with 1% TTC solution at 37°C for 15 min. Red parts in the heart stained by TTC indicated ischemic but viable tissue. However, staining negative areas represented the infarcted myocardium. Areas of infarct size were measured by computerized planimetry. The size of infarction area was expressed as a percentage of the total left ventricular area.

### 2.3. Histopathologic Examination

The heart samples were quickly removed and fixed in 10% formalin for 24 h, then sliced into pieces of 5 *μ*m thick, and stained with hematoxylin-eosin. Slides were imaged under a light microscope.

### 2.4. Measurement of Serum Lactate Dehydrogenase and Creatine Kinase

The detection kits of lactate dehydrogenase (LDH, A020-2-2) and creatine kinase (CK, A032-1-1) were from Nanjing Jiancheng Bioengineering Institute. At the end of 24 h of reperfusion, blood samples from each group were collected. And serum supernatant was obtained by centrifugation at 3500 rpm for 10 min. The supernatant was immediately stored at -70°C until analysis. All procedures were performed following the manufacturer's instructions, respectively.

### 2.5. Determination of Malondialdehyde, Glutathione Peroxidase, and Reactive Oxygen Species *In Vivo*

The detection kits of malondialdehyde (MDA, A003-1-2) and glutathione peroxidase (GSH-Px, A005-1-2) were purchased from Nanjing Jiancheng Bioengineering Institute. The activities of MDA and GSH-Px in serum were evaluated following the manufacturer's instructions, respectively. As for measurement of reactive oxygen species (ROS), the samples of the left ventricular myocardium were incubated with the DHE (5 *μ*mol/L, S0063, Beyotime) at 37°C for 30 min, then washed with PBS for 3 times. The fluorescence intensity was examined using a confocal scanning microscope (LSM700, Zeiss, USA), and all images were analyzed using Image-Pro Plus software. And for each slice, 3 fields were randomly obtained for quantification.

### 2.6. TUNEL Staining

TUNEL BrightGreen apoptosis detection kit (A112-01) was obtained from Vazyme Biotech. Briefly, the heart samples were collected and fixed in 4% paraformaldehyde solution for 30 min. Then, apoptotic cells were measured following the manufacturer's instructions. Apoptotic nuclei and total cardiomyocyte nuclei were labeled with green fluorescein staining and DAPI, respectively. For each slice, they were randomly collected under with an LSM700 confocal microscope. The extent of cell apoptosis was expressed as the ratio of TUNEL-positive nuclei over DAPI-stained nuclei.

### 2.7. Immunohistochemistry of Cleaved Caspase-3

The heart samples were cut into 5 *μ*m thick sections, deparaffinized in xylene, and rehydrated in PBS. Endogenous peroxidase activity was blocked with 3% hydrogen peroxide in methanol for 30 min. The slides were then incubated overnight at 4°C in a humid chamber with anti-cleaved caspase-3 antibody (dilution 1 : 100, 9661S, Cell Signaling Technology). After washing for three times, the slides were incubated with the HRP-conjugated secondary antibody (dilution 1 : 200, BS13278) at 37°C for 1 h. After incubation with DAB and counterstaining with hematoxylin, a light microscope was applied to examine the dehydrated sections. All images were analyzed using Image-Pro Plus software. For each slice, 3 fields were randomly obtained for quantification.

### 2.8. Cell Culture, Cell Viability, and H/R Injury Model *In Vitro*

The rat H9c2 cardiomyocyte cell line in our study was obtained from Shanghai Institute of Cell Biology, Chinese Academy of Sciences (Shanghai, China). The H9c2 cells were maintained in DMEM supplemented with 10% FBS, penicillin (100 U/mL), and streptomycin (100 *μ*g/mL). All cells were incubated at 37°C in a humidified atmosphere of 5% CO_2_. The effects of schisandrol A (SA, 14-56 *μ*mol/L, lot number: 16103103, Chengdu Pufei De Biotech) on cell viability were measured by using methylthiazolyldiphenyl-tetrazolium bromide (MTT, ST316, Beyotime). In order to mimic the MI/R injury *in vitro*, the hypoxia/reoxygenation (H/R) model was established as a previous protocol described [19]. In present experiment, cells were treated with SA or N-acetyl cysteine (NAC, 500 *μ*mol/L, A9165, Sigma-Aldrich) during the whole H/R process. For H/R experiments, the H9c2 cells were rinsed twice with serum-free and glucose-free DMEM and subjected to a hypoxic environment of 94% N_2_, 5% CO_2_, and 1% O_2_ at 37°C for 6 h. Subsequently, the cardiomyocytes were reoxygenated by return to a standard incubator with 5% CO_2_ in normal atmosphere at 37°C for an additional 6 h.

### 2.9. Measurement of LDH, MDA, and ROS *In Vitro*

After H/R injury, the culture supernatants were obtained and the release of LDH was detected following the manufacturer's instructions. Meanwhile, the supernatants of cell lysates from each group were collected after H/R injury and the level of MDA was measured following the manufacturer's instructions. As for intracellular ROS, ROS assay kit (S0033S, Beyotime) was used in the experiment. In short, H9c2 cardiomyocytes were washed with PBS for three times after H/R injury and then exposed to serum-free medium containing 10 *μ*mol/L DCFH-DA probe and incubated in the dark at 37°C for 30 min. Then, the cells were washed and examined under a fluorescence microscope.

### 2.10. Hoechst 33342 Staining and Flow Cytometric Analysis

Hoechst 33342 detection kit (C1026) and Annexin V-FITC apoptosis detection kit (C1062L) were purchased from Beyotime. After H/R injury, 5 *μ*g/mL Hoechst 33342 reagent was added to H9c2 cardiomyocytes and incubated for 30 min in the dark at 37°C, followed by the addition of 15 *μ*g/mL PI and incubation for 30 min in the dark at 4°C. Then, cells were washed with PBS for three times and imaged using a fluorescence microscope. The nucleus of apoptotic cells was irregular and hypercoagulable (bright staining). Further, double staining with Annexin V-FITC and PI was carried out to identify apoptotic cells. After H/R injury, the cells were collected and washed with PBS. Then, cells were resuspended with binding buffer and incubated with Annexin V-FITC and PI at room temperature for 15 min in the dark. Cellular fluorescence was measured with a flow cytometer (Becton Dickinson, USA).

### 2.11. *In Vitro* Plasmid and siRNA Transfection

The HEK 293T cells were seeded onto six-well plates and cultured for 24 h prior to transient transfection. Then, cells were transfected at 70-80% confluence with 14-3-3*θ* plasmid and negative control plasmid using the EndoFectin transfection reagent. After transfection for 48 h, the HEK 293T cells were collected and the whole-cell extracts were analyzed in subsequent experiments. Meantime, H9c2 cells were transfected with siRNA against 14-3-3*θ* or scrambled siRNA as a control. In brief, the cells were transfected with 100 nmol/L siRNA following the manufacturer's instructions. The 14-3-3*θ* siRNA transfection efficiency was determined by western blot analysis with *β*-actin as a reference.

### 2.12. Western Blot Analysis

The protein concentration was calculated using a BCA protein assay kit (P0012, Beyotime) following the manufacturer's instructions. Equal amounts of total protein (40 *μ*g) were electrophoresed on 12.5% SDS-PAGE gels and transferred onto PVDF membranes. The membranes were then blocked with 5% BSA and incubated overnight at 4°C with specific primary antibodies: 14-3-3*θ* (dilution 1 : 200, sc-59414, Santa Cruz), cleaved caspase-3 (dilution 1 : 1000), Bax (dilution 1 : 1000, ab32503, Abcam), and Bcl-2 (dilution 1 : 1000, 26593-1-AP, Proteintech). The blots were probed with peroxidase conjugated secondary antibody at a 1 : 8000 dilution. The protein-antibody complexes were subsequently detected with ECL plus system and visualized by ChemiDoc™ MP System (Bio-Rad) and analyzed using Image Lab™ Software (version 4.1, Bio-Rad).

### 2.13. Immunofluorescence Staining (IF)

The heart sections with 10 *μ*m thick transverse or cultured cells were washed with ice-cold PBS, fixed in 4% paraformaldehyde, permeabilized with 0.1% Triton X-100 in PBS for 30 min, blocked with 5% BSA, and incubated with the primary antibody against cleaved caspase-3 (dilution 1 : 200) overnight at 4°C. Sections were washed repeatedly and then incubated with secondary antibody (dilution 1 : 300, BS10018, Bioworld) for 2 h. DAPI was used to stain the cell nuclei. The fluorescence intensity was examined under a confocal laser scanning microscope.

### 2.14. Capture and Identification of Target Proteins Using Serial Affinity Chromatography

SA affinity matrices were performed as previously described [20]. Heart proteins from MI/R model were collected. Heart lysates were incubated with SA-EAS 6B beads at 4°C with gentle shaking for 4 h in bead buffer and then centrifuged at 8000 rpm for a short period. Then, another 60 *μ*L SA-EAS 6B beads was added to the supernatant and swung gently for additional 4 h, finally centrifuged. Next, the two portions of the resulting matrices were washed twice, respectively, with bead buffer, resuspended in SDS-PAGE sample buffer, boiled, and finally centrifuged. The supernatants were, respectively, subjected to SDS-PAGE analysis and confirmed by western blot analysis.

### 2.15. Molecular Docking

The Molecular Operating Environment (MOE, version 2009.10) program was used to prepare the complex structure of the ligand and 14-3-3*θ*. All ligand molecules and water molecules were removed. The missing hydrogen atoms of the crystal structure of 1 in complex with 14-3-3*θ* were added, and protonate 3D module was used to predict the protonation state of the acidic and basic amino acid residues. The induced fit docking (IFD) method was used to dock SA into the identified allosteric sites of 14-3-3*θ* by Glide 7.0. And energy minimized using LigPrep with the OPLS-2005 force field and the ionized state at a pH value of 7.0 ± 2.0 was assigned by Epik. The receptor structure preparation was accomplished by the Protein Preparation Wizard. The center of grid box was set to coordinate with allosteric sites predicted by PARS, and the inner grid was sized at 18 × 18 × 18 Å3 to include the overlap region detected simultaneously by Corriste and Sitemap. In the IFD flow, the standard precision was first adopted, in which the van der Waals scaling values of both the receptor and the ligand were set to 0.5 and the maximum number of poses was set to 30. Then, residues within 5.0 Å of the ligand were set to be flexible in the process of prime refinement. Finally, 20 top-ranked poses were output after redocking.

### 2.16. MST Analysis

The interaction of SA with protein 14-3-3*θ* was detected following the MST procedure. Briefly, SA was diluted from 1 mmol/L to 0.0625 nmol/L in ddH_2_O and 14-3-3*θ* protein was diluted to 40 nmol/L in PBS. Then, SA and 14-3-3*θ* were mixed in a 1 : 1 volumetric ratio and incubated for 5 min at room temperature in dark and finally analyzed by NanoTemper Monolith NT.115 machine via MO.Affinity Analysis software provided by NanoTemper.

### 2.17. Statistical Analysis

All values are expressed as the mean ± SD. The data sets from individual experiments were statistically analyzed using Student's two-tailed *t*-test for comparisons between two groups and one-way analysis of variance (ANOVA) followed by Dunnett's test for comparisons between three or more groups. *P* < 0.05 was considered statistically significant.

## 3. Results

### 3.1. SA Attenuated Myocardial Injury in MI/R Mice

**Figure 1:**
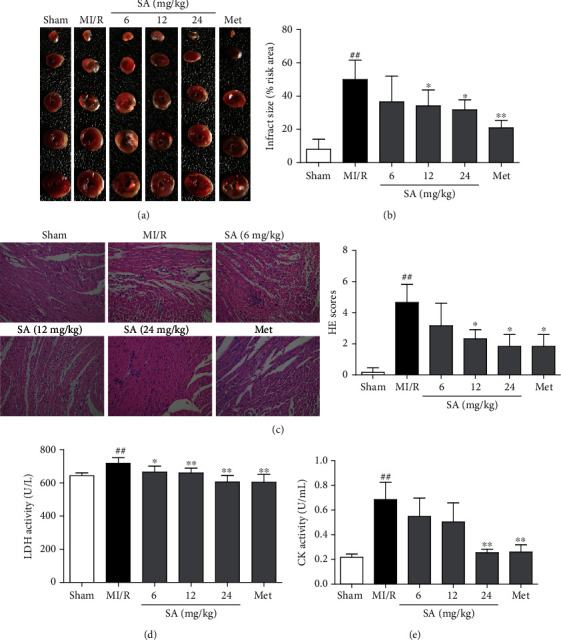
SA attenuated myocardial injury in MI/R mice. (a) TTC staining of the myocardial infarct area. (b) Graphic representation of the myocardial infarct size. (c) Representative images of the myocardium measured by HE staining (200x magnification). (d) LDH activity. (e) CK activity. The data were expressed as the mean ± SD. ^##^*P* < 0.01 vs. sham group; ^∗^*P* < 0.05 and ^∗∗^*P* < 0.01 vs. MI/R group (*n* = 6).

To investigate the protective effect of SA against MI/R-induced injury *in vivo*, the myocardial infarct size, histopathological examination, and some serum biochemical indicators were detected. As shown in Figure 1, 30 min of ischemia and 24 h of reperfusion resulted in myocardial injury, as reflected by increased infarct size, morphological alterations, and increased LDH and CK activities, whereas SA at the dosage of the 12-24 mg/kg treatment group markedly inhibited the increase of myocardial infarct size, which exhibited the same protection effect as the positive drug Met (Figures 1(a) and 1(b)). In addition, the results of histopathological examination revealed widespread myocardial structural disorder, increased necrosis, and a large number of inflammatory cells infiltrating the myocardial tissue, while treatment of SA or Met markedly ameliorated histological features in myocardial tissue (Figure 1(c)). Moreover, SA could significantly reduce the activities of LDH and CK in serum (Figures 1(d) and 1(e)). Collectively, these data indicated that SA showed an obvious protection in mice with MI/R injury.

### 3.2. SA Decreased Oxidative Stress in MI/R Mice

**Figure 2:**
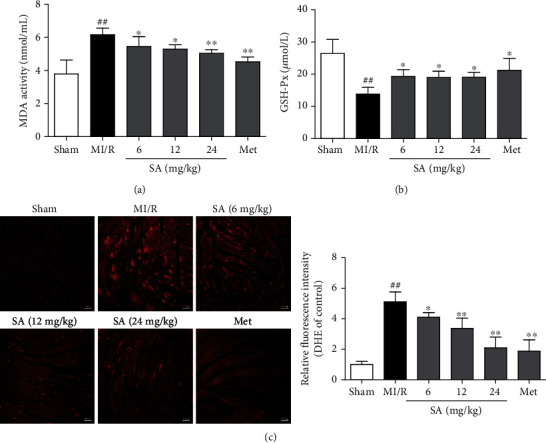
SA decreased oxidative stress in MI/R mice. (a) MDA activity. (b) GSH-Px content. (c) Representative images of DHE staining (bar = 50 *μ*m). The data were expressed as the mean ± SD.^##^*P* < 0.01 vs. sham group; ^∗^*P* < 0.05 and ^∗∗^*P* < 0.01 vs. MI/R group (*n* = 6).

To confirm the vital role of antioxidation of SA in resisting MI/R injury, oxidative stress-related molecules such as MDA, GSH-Px, and ROS were detected. As shown in Figure 2(a), the levels of MDA profoundly increased after MI/R injury but decreased after treatment with SA and Met, when compared with the untreated MI/R group. And higher GSH-Px was observed after the treatment of SA at the dosage of 6-24 mg/kg as well as Met than that in the untreated MI/R group (Figure 2(b)). As indicated in Figure 2(c), the levels of ROS were significantly higher in the MI/R group than those in the sham group, whereas these were significantly reduced after SA and Met treatment. Taken together, SA was demonstrated to exert an apparent cardioprotective effects against MI/R injury by inhibiting oxidative stress injury.

### 3.3. SA Inhibited Cardiomyocyte Apoptosis in MI/R Mice

**Figure 3:**
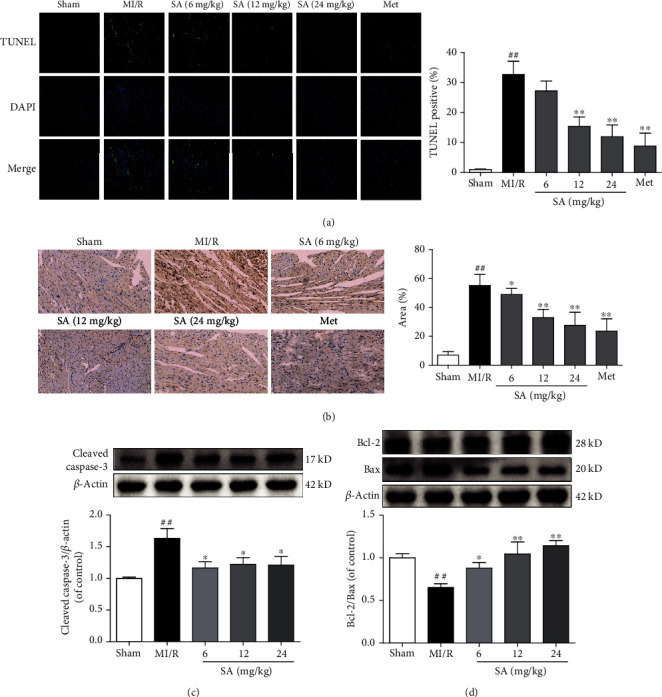
SA inhibited cardiomyocyte apoptosis in MI/R mice. (a) Representative images of TUNEL staining (bar = 10 *μ*m). (b) Immunohistochemical staining of cleaved caspase-3 (bar = 100 *μ*m). (c) The expression of cleaved caspase-3 was detected by western blot. (d) The expressions of Bcl-2 and Bax were detected by western blot. The data were expressed as the mean ± SD. ^##^*P* < 0.01 vs. sham group; ^∗^*P* < 0.05 and ^∗∗^*P* < 0.01 vs. MI/R group (*n* = 3).

Following MI/R, the number of TUNEL-positive (apoptotic) cells markedly increased compared with the sham-treated mice, whereas a notable decrease of TUNEL-positive rate was observed in the SA and Met treatment groups (Figure 3(a)). In addition, immunohistochemical results exhibited that SA and Met administration profoundly decreased the expression of cleaved caspase-3 in MI/R mice (Figure 3(b)). Moreover, as illustrated in Figures 3(c) and 3(d), the MI/R mice obviously increased cleaved caspase-3 expression and decreased the ratio of Bcl-2/Bax, compared with the sham-treated mice. In contrast, a downregulation of cleaved caspase-3 expression and upregulation of Bcl-2/Bax ratio were observed from SA-treated mice. These results demonstrated a potent antiapoptotic effect of SA in MI/R injury.

### 3.4. SA Attenuated H9c2 Cardiomyocyte Injury and Oxidative Stress Induced by H/R

**Figure 4:**
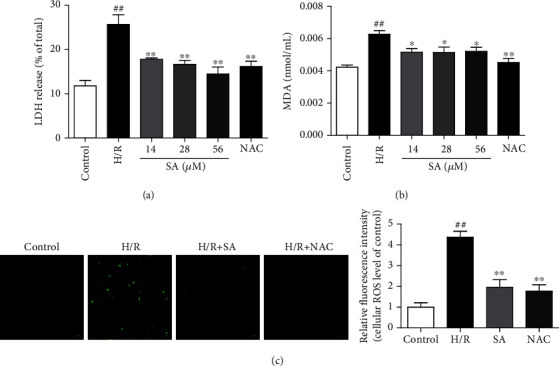
SA attenuated H9c2 cardiomyocyte injury and oxidative stress induced by H/R. H9c2 cells were treated with SA and then exposed to hypoxia of 6 h followed by 6 h reoxygenation. (a) LDH release. (b) MDA content. (c) Representative images of ROS level in H9c2 cardiomyocytes (bar = 50 *μ*m). The data were expressed as the mean ± SD. ^##^*P* < 0.01 vs. control group; ^∗^*P* < 0.05 and ^∗∗^*P* < 0.01 vs. H/R group.

To further confirm the protection of SA, an *in vitro* experiment by using H9c2 cells with H/R induction was performed. Firstly, the cell viability after treatment with SA was examined and the results showed that SA at concentrations of 14-56 *μ*mol/L did not markedly affect the viability (Figure S1(a)). Exposure of cardiomyocytes to H/R resulted in a significant decrease in cell viability and morphological changes, while treatment with 14-56 *μ*mol/L SA following H/R induction increased cell viability, improved the cell adherence, and maintained the cell morphology (Figure S1(b) and S1(c)). As LDH release is a recognized important marker of myocardial cell damage, the release of LDH was also detected. The results showed that the increased release of LDH in H/R-induced cardiomyocyte injury significantly reduced after treatment with SA (Figure 4(a)). Meanwhile, SA significantly decreased the levels of MDA (Figure 4(b)). In addition, intracellular ROS was further reflected by DCFH-DA fluorescence probe. The levels of intracellular ROS in cardiomyocytes undergoing H/R injury significantly decreased after treatment with SA. These results indicated that SA could alleviate H/R-induced cardiomyocyte injury and decrease oxidative stress damage.

### 3.5. SA Inhibited H9c2 Cardiomyocyte Apoptosis Induced by H/R

**Figure 5:**
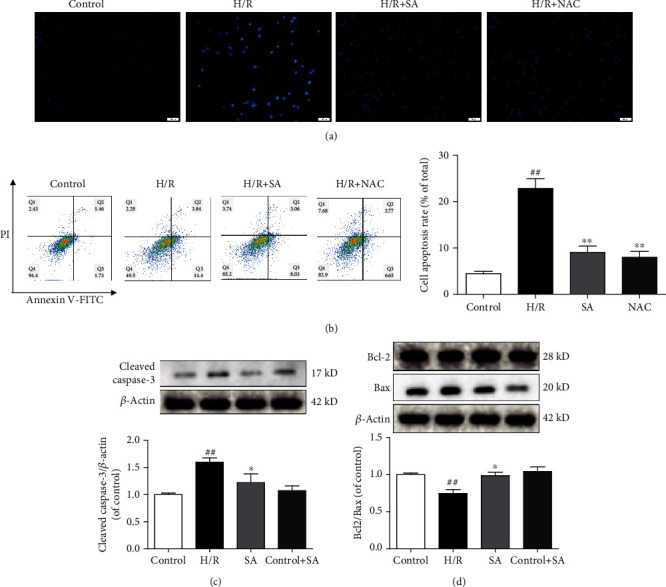
SA inhibited H9c2 cardiomyocyte apoptosis induced by H/R. H9c2 cells were treated with SA and then exposed to hypoxia of 6 h followed by 6 h reoxygenation. (a) Representative images of Hoechst 33342 staining (bar = 50 *μ*m). (b) Cell apoptosis was detected by flow cytometry analysis. (c) The expression of cleaved caspase-3 was detected by western blot. (d) The expressions of Bcl-2 and Bax were detected by western blot. The data were expressed as the mean ± SD. ^##^*P* < 0.01 vs. control group; ^∗^*P* < 0.05 and ^∗∗^*P* < 0.01 vs. H/R group.

The protective effects of SA on H/R-induced apoptosis were confirmed by Hoechst 33342 staining. The results showed that H/R injury induced apoptosis in H9c2 cells, as evidenced by chromatin condensation and fragmentation, the typical features of apoptotic cells. However, SA treatment restored cell nuclei to normal morphology and significantly reduced nuclear condensation and fragmentation of H9c2 (Figure 5(a)). Quantitative analysis using flow cytometric assay further validated that SA treatment markedly decreased the apoptotic index (Figure 5(b)). As illustrated in Figures 5(c) and 5(d), SA treatment notably reduced the expression of cleaved caspase-3 protein but increased the ratio of Bcl-2/Bax. In summary, these results demonstrated SA treatment exerted the protective effect against cardiomyocyte apoptosis induced by H/R.

### 3.6. SA Ameliorated Myocardial Injury by Enhancing 14-3-3*θ* Level

To further confirm the underlying mechanism of SA, serial affinity chromatography, molecular docking, and MST were performed to capture and identify the potential target proteins. Through the SA affinity probe, specific binding proteins for SA of MI/R mouse heart tissue were captured by serial affinity chromatography as shown in Figure S2(a). The amounts of proteins A/B, as indicated in the SDS-PAGE, markedly decreased following the serial affinity chromatography, suggesting that the reduced proteins might be the specific SA-binding proteins. Based on the previous network pharmacology and experimental studies [21, 22], we further confirmed that 14-3-3*θ* protein might be the binding target protein of SA, according to the results of western blot. Additionally, molecular docking was applied to predict the binding modes of 14-3-3*θ* and SA. As shown in Figure S2(b)-(c), the activity domain between 14-3-3*θ* and SA was screened, and the possible amino acid positions were selected. Moreover, the MST method was applied to detect the binding affinity between SA and the protein 14-3-3*θ*. The results exhibited that SA has a certain combination with 14-3-3*θ*, and its dissociation constant *Kd* value was 206.7 ± 9.615 *μ*mol/L (Figure S2(d)).

**Figure 6:**
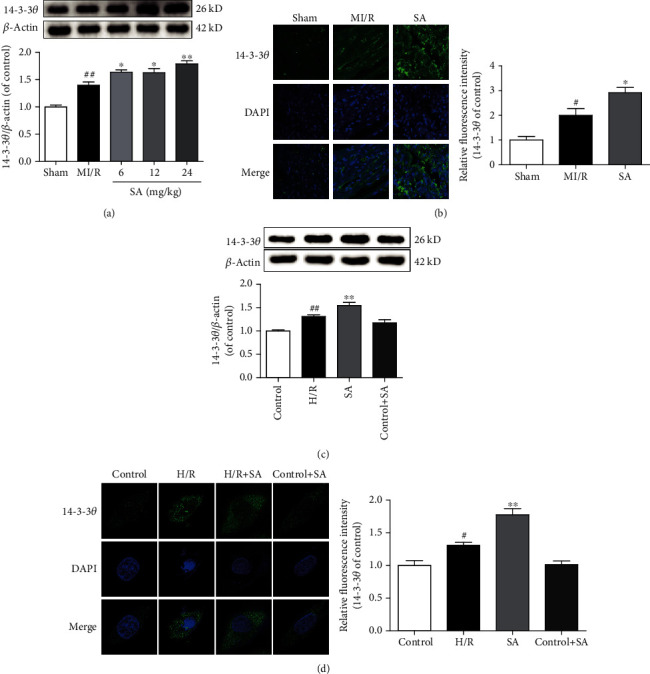
SA ameliorated myocardial injury by enhancing 14-3-3*θ*. (a) The expression of 14-3-3*θ* was detected by western blot in MI/R mice. (b) Representative immunofluorescence images of 14-3-3*θ* in MI/R mice (bar = 20 *μ*m). (c) The expression of 14-3-3*θ* was detected by western blot in H/R-induced cardiomyocyte injury. (d) Representative immunofluorescence images of 14-3-3*θ* in H/R-induced cardiomyocyte injury. The data were expressed as the mean ± SD. ^##^*P* < 0.01 and ^#^*P* < 0.05 vs. sham or control group; ^∗^*P* < 0.05 and ^∗∗^*P* < 0.01 vs. MI/R or H/R group.

Subsequently, we further investigated whether 14-3-3*θ* was involved in the cardioprotection of SA against MI/R injury and H/R injury. As shown in Figures 6(a) and 6(b), the expression of 14-3-3*θ* significantly increased after MI/R injury. Interestingly, treatment with the SA markedly further increased 14-3-3*θ* protein expression. As indicated in Figures 6(c) and 6(d), exposure of cells to H/R led to an obvious increase in 14-3-3*θ* expression, which was significantly ameliorated by SA treatment. These results revealed that SA might enhance the expression of 14-3-3*θ* to inhibit myocardial apoptosis after MI/R and H/R injury.

### 3.7. 14-3-3*θ* Protein Exerted an Essential Role in H/R-Induced Cardiomyocyte Apoptosis

**Figure 7:**
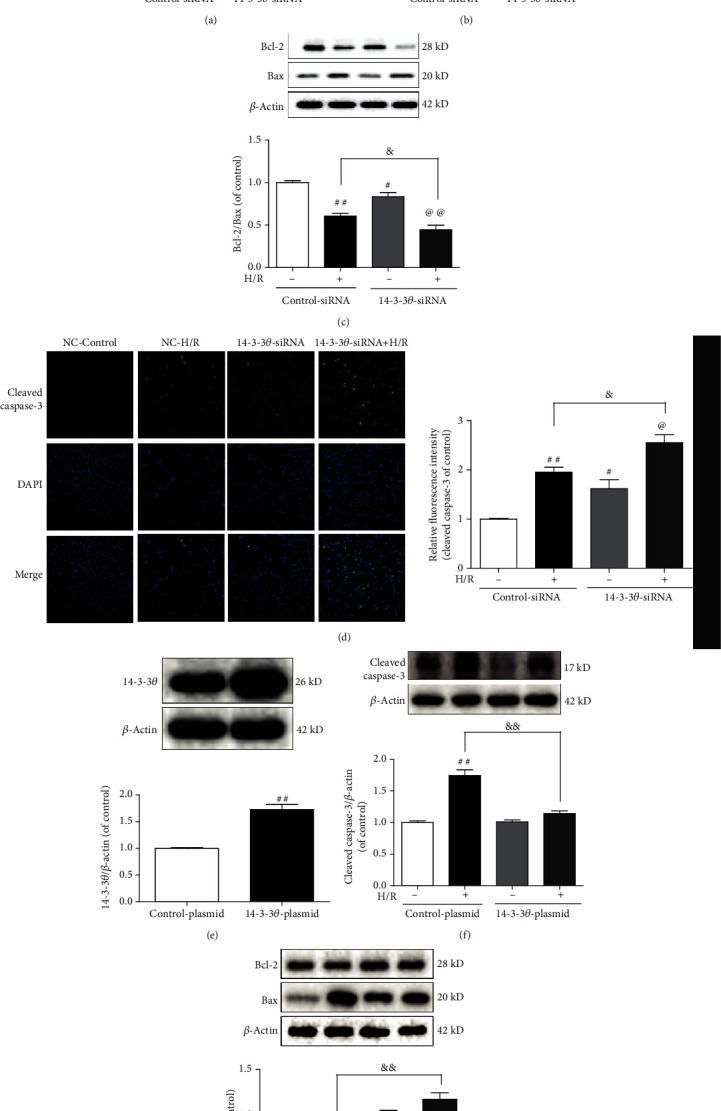
14-3-3*θ* protein exerts an essential role in H/R-induced cardiomyocyte apoptosis. (a) The efficiency of 14-3-3*θ* siRNA was detected by western blot. (b) The expression of cleaved caspase-3 was detected by western blot. (c) The expressions of Bcl-2 and Bax were detected by western blot. (d) Representative immunofluorescence images of cleaved caspase-3 (bar = 50 *μ*m). (e) The efficiency of 14-3-3*θ* overexpression was detected by western blot. (f) The expression of cleaved caspase-3 was detected by western blot. (g) The expressions of Bcl-2 and Bax were detected by western blot. The data were expressed as the mean ± SD. ^##^*P* < 0.01 and ^#^*P* < 0.05 vs. control siRNA/plasmid group; ^@^*P* < 0.05 and ^@@^*P* < 0.01 vs. 14-3-3*θ* siRNA/plasmid group; ^&^*P* < 0.05 and ^&&^*P* < 0.01 vs. control siRNA/plasmid+H/R group.

Previous studies have confirmed that 14-3-3*η* and 14-3-3*γ* proteins participated in acute myocardial injury [14, 15]. To further confirm the effects of 14-3-3*θ* on cardiomyocyte apoptosis, we evaluated the effects of knockdown or overexpression of 14-3-3*θ* in a H/R-induced cell model. As shown in Figure 7(a), siRNA-mediated knockdown of 14-3-3*θ* gene was successfully conducted with the inhibition efficacy larger than 50%. As illustrated in Figures 7(b)–7(d), interfering 14-3-3*θ* further aggravated the apoptosis of cardiomyocytes after H/R injury, as reflected by the increased expression of cleaved caspase-3 and the reduced ratio of Bcl-2/Bax. All these results indicated that 14-3-3*θ* interference could promote cardiomyocyte apoptosis and aggravate myocardial injury. In addition, as shown in Figure 7(e), the overexpression efficiency of 14-3-3*θ* in 293T cells reached over 60%. Furthermore, 14-3-3*θ* overexpression in 293T cells measurably reduced cleaved caspase-3 expression and increased Bcl-2/Bax ratio (Figures 7(f) and 7(g)), which suggested that 14-3-3*θ* overexpression could profoundly attenuate the H/R-induced apoptosis. Taken together, 14-3-3*θ* played a vital role in the protection of MI/R injury and served as a potential therapeutic target.

### 3.8. SA Alleviated H/R-Induced H9c2 Cardiomyocyte Apoptosis Partly through 14-3-3*θ*

**Figure 8:**
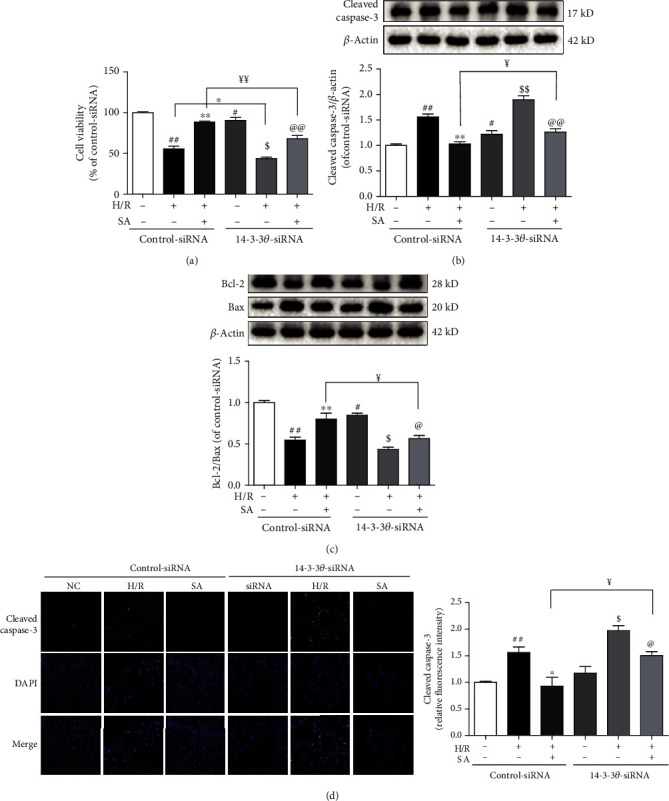
SA alleviated H/R-induced H9c2 cardiomyocyte apoptosis partly through 14-3-3*θ*. (a) The cell viability was detected by MTT assay. (b) The expression of cleaved caspase-3 was detected by western blot. (c) The expressions of Bcl-2 and Bax were detected by western blot. (d) Representative immunofluorescence images of cleaved caspase-3 (bar = 50 *μ*m). The data were expressed as the mean ± SD. ^##^*P* < 0.01 vs. control siRNA group; ^∗∗^*P* < 0.01 vs. control siRNA+H/R group; ^$^*P* < 0.05 and ^$$^*P* < 0.01 vs. 14-3-3*θ* siRNA group; ^@^*P* < 0.05 and ^@@^*P* < 0.01 vs. 14-3-3*θ* siRNA+H/R group; ^￥^*P* < 0.05 and ^￥￥^*P* < 0.01 vs. control siRNA+H/R+SA group.

As illustrated in Figure 8(a), compared with the control-siRNA+H/R+SA group, cell viability of the 14-3-3*θ*-siRNA+H/R+SA group significantly decreased, which indicated that 14-3-3*θ*-siRNA partly weakened the cardioprotection of SA during H/R injury. Furthermore, as shown in Figures 8(b)–8(d), 14-3-3*θ*-siRNA partially suppressed downregulation of cleaved caspase-3 expression and also partly inhibited upregulation of Bcl-2/Bax ratio caused by SA. All these data indicated that SA could exert significant cardioprotection against H/R injury via inhibition of myocardial apoptosis, at least in part, through upregulating of 14-3-3*θ*.

## 4. Discussion

In this study, SA treatment obviously reduced myocardial infarct size, ameliorated cardiac pathological alterations, improved cell viability, and decreased activities of LDH and CK. Further experiments confirmed that SA exerted antioxidation effect by regulating MDA, ROS, and GSH-Px levels or activities, prevented cell apoptosis as evidenced by decreased TUNEL-positive rate and cleaved caspase-3 expression, and increased Bcl-2/Bax ratio. Additionally, SA obviously elevated 14-3-3*θ* expression, thereby attenuating cardiac damage in MI/R or H/R injury. Silencing 14-3-3*θ* decreased the protection of SA in H/R-induced cell injury via increasing cleaved caspase-3 expression and decreasing the Bcl-2/Bax ratio. Collectively, these data demonstrated that SA is an effective treatment which ameliorated myocardial damage by upregulating 14-3-3*θ* in MI/R-induced cardiomyocyte apoptosis.

Myocardial I/R injury is a serious condition characterized by the excess free radicals deposition which often contributes to oxidation, denaturation, and degradation of nucleic acids, protein, and polysaccharide molecules, and ultimately results in myocardial apoptosis and death [23]. Reperfusion is correlated with a burst of ROS production, which exerts a critical role in myocardial damage [24]. High ROS can impair cardiac contractile function and induce cell apoptosis, which eventually led to myocardial remodeling or heart failure. Thus, inhibiting oxidative stress damage prevents MI/R injury [23], consistent with these studies that MI/R or H/R led to an increase of oxidative stress as illustrated by the higher MDA and ROS and lower GSH-Px levels, whereas a significant increase of GSH-Px level and decrease of MDA and ROS levels were observed after SA treatment. Thus, SA treatment showed a beneficial protection on MI/R injury through inhibiting oxidative stress damage.

An increasing number of literatures demonstrate that the dropout of cardiomyocytes induced by apoptosis is crucial in multiple heart diseases and inevitably develop into heart failure [25]. Apoptosis occurs in myocytes during MI/R injury and regards as a major cell death mechanism in ischemia/reperfusion injury [26, 27]. Apoptosis induced by oxidative stress accelerates the loss of cardiomyocytes and inhibits the recovery of cardiac function [25]. Prior studies showed that MI/R injury led to cell apoptosis, activation of caspase-3, and disruption of balance between anti- and proapoptotic proteins in Bcl-2 family proteins [28]. It has been reported that Bcl-2 has been localized to the mitochondria, endoplasmic reticulum, and nuclear membranes and also the sites of reactive oxygen species generation, which could inhibit the formation of Bax on mitochondrial membrane [29]. The ratio of Bcl-2/Bax determines the survival or death of cells following an apoptotic stimulus [30]. In addition, caspase cascades are crucial triggers of apoptosis. Among them, caspase-3 is a common activated death protease, catalyzing the specific cleavage of multiple important cellular proteins. As one of the key effectors, caspase-3 is essential for the implementation of the final step of apoptosis [31]. Targeting cell apoptosis has been proved to be a promising therapeutic approach to ameliorate or even inhibit myocardial injury. Our results showed that SA decreased the TUNEL-positive rate and cleaved caspase-3 expression and increased the Bcl-2/Bax ratio, which demonstrated an obvious inhibition of cell apoptosis by SA. Thus, SA treatment showed a beneficial protection on MI/R injury through inhibiting apoptosis.

The 14-3-3 protein family expressed in most tissues is a conserved regulatory molecule, owning the ability to bind a multitude of functionally diverse signaling proteins and exert a significant role in regulating signal transduction, stress response, and apoptosis [32]. The 14-3-3 proteins sequester phosphorylated BAD in the cytoplasm and deter it from associating with Bcl-xL, and eventually inhibit apoptosis [33]. Previous studies have provided that 14-3-3 proteins could inhibit apoptosis through regulating MAPK cascades involving the JNK1 and p38 MAPKs [34]. Recent studies have reported that preconditioning exercise reduces brain damage and neuronal apoptosis through enhanced endogenous 14-3-3*γ* after focal brain ischemia in rats [35]. Additionally, 14-3-3*γ* has been found to mediate the inhibitory effect of curcumin and quercetin on cardiotoxicity induced by doxorubicin through inhibiting oxidative stress and protecting against mitochondrial dysfunction [36, 37]. Tetramethylpyrazine was also verified to attenuate doxorubicin-induced endotheliotoxicity and mitochondrial dysfunction through 14-3-3*γ*/Bcl-2 [38]. Moreover, inhibition of 14-3-3 facilitates dopaminergic neuron loss and overexpression of 14-3-3*θ* accelerates recovery in the MPTP mouse model of Parkinson's disease [39]. Despite extensive research, the function of the 14-3-3*θ* protein is still not completely understood in MI/R injury.

On the basis of the obtained results that SA protected cardiomyocytes against apoptosis *in vivo* and *in vitro*, further experiments to identify potential target of SA were conducted. The serial affinity chromatography method was carried out to confirm the possible target of SA [20–22]. Furthermore, molecular docking and MST method were performed for further analysis. These results showed that SA had some certain binding capability to the key functional protein 14-3-3*θ*. Based on these findings, the underlying mechanism involved in the antiapoptotic effects of SA was conducted. In the present study, the expression of 14-3-3*θ* was decreased in both H/R-treated H9c2 and MI/R mice, whereas enhanced 14-3-3*θ* by SA obviously ameliorated oxidative damage and apoptosis. Specific interference with 14-3-3*θ* could aggravate cardiomyocyte apoptosis induced by H/R injury, whereas overexpression of 14-3-3*θ* in 293T cell could significantly inhibit the cell apoptosis. SA exerted the ability to upregulate the expression of 14-3-3*θ*, and 14-3-3*θ* siRNA knockdown weakens its protection against H/R-induced cardiomyocyte apoptosis, indicating that 14-3-3*θ* upregulation was essential for its function in the MI/R injury. These data indicated that 14-3-3*θ* played a key role in the protection of SA against MI/R-induced myocardial apoptosis. However, this study also has some limitations. We revealed the functions of 14-3-3*θ* in H/R-induced cardiomyocyte injury, but the potential mechanisms need to be further explored. The signaling pathways involved in the protective effect of SA through key functional protein 14-3-3*θ* remain to be further investigated. Although we have demonstrated that upregulation of 14-3-3*θ* might ameliorate MI/R injury, further validation is required using 14-3-3*θ*-deficient mice. Moreover, this study does not exclusively eliminate other potential targets that might play an important role in the cardiac protection of SA.

## 5. Conclusions

Taken together, we have confirmed that SA protected the myocardium against MI/R injury and suppressed MI/R-induced oxidative stress and myocardial apoptosis. More importantly, we found that SA showed some ability to bind with 14-3-3*θ* protein. And upregulation of 14-3-3*θ* inhibits myocardial apoptosis and might attenuate MI/R injury. All these results demonstrated that the cardioprotective effect of SA exerted at least partially through upregulation of 14-3-3*θ* to inhibit myocardial apoptosis. These findings indicated that 14-3-3*θ* might be a potential therapeutic target for MI/R injury treatment and also might lay the foundation for further development of new drug for cardiovascular diseases.

## Data Availability

No data were used to support this study.

## Supplementary Materials

Supplementary Materials The effects of SA on cell viability were evaluated by MTT with or without H/R exposure. Figure S1: SA protected cardiomyocytes against injury induced by H/R. H9c2 cells were treated with SA and then exposed to hypoxia of 6 h followed by 6 h reoxygenation. (a) Effect of SA on H9c2 cardiomyocytes. (b) Cell viability. (c) Representative images of cell morphology. The target protein of SA was screened and identified using serial affinity chromatography, molecular docking, and MST analysis. In addition, the possible binding sites were screened out by software analysis. Figure S2: target screening and validation of SA. (a) Capture and identification of target proteins using serial affinity chromatography. (b) Molecular docking of 14-3-3*θ* and SA. (c) Software analysis was performed to screen out possible amino acids for interaction. (d) The combining capability between SA and 14-3-3*θ* protein was detected by MST analysis.
