# Supplementary material for: Schisandrol A Attenuates Myocardial Ischemia/Reperfusion-Induced Myocardial Apoptosis through Upregulation of 14-3-3θ
Source: Oxid Med Cell Longev. 2021 Jun 26;2021:5541753. doi: 10.1155/2021/5541753 (PMC8257380; doi:10.1155/2021/5541753)
Supplement: Supplementary Materials — The effects of SA on cell viability were evaluated by MTT with or without H/R exposure. Figure S1: SA protected cardiomyocytes against injury induced by H/R. H9c2 cells were treated with SA and then exposed to hypoxia of 6 h followed by 6 h reoxygenation. (a) Effect of SA on H9c2 cardiomyocytes. (b) Cell viability. (c) Representative images of cell morphology. The target protein of SA was screened and identified using serial affinity chromatography, molecular docking, and MST analysis. In addition, the possible binding sites were screened out by software analysis. Figure S2: target screening and validation of SA. (a) Capture and identification of target proteins using serial affinity chromatography. (b) Molecular docking of 14-3-3θ and SA. (c) Software analysis was performed to screen out possible amino acids for interaction. (d) The combining capability between SA and 14-3-3θ protein was detected by MST analysis. [file 5541753.f1.pdf]

Supplementary Materials

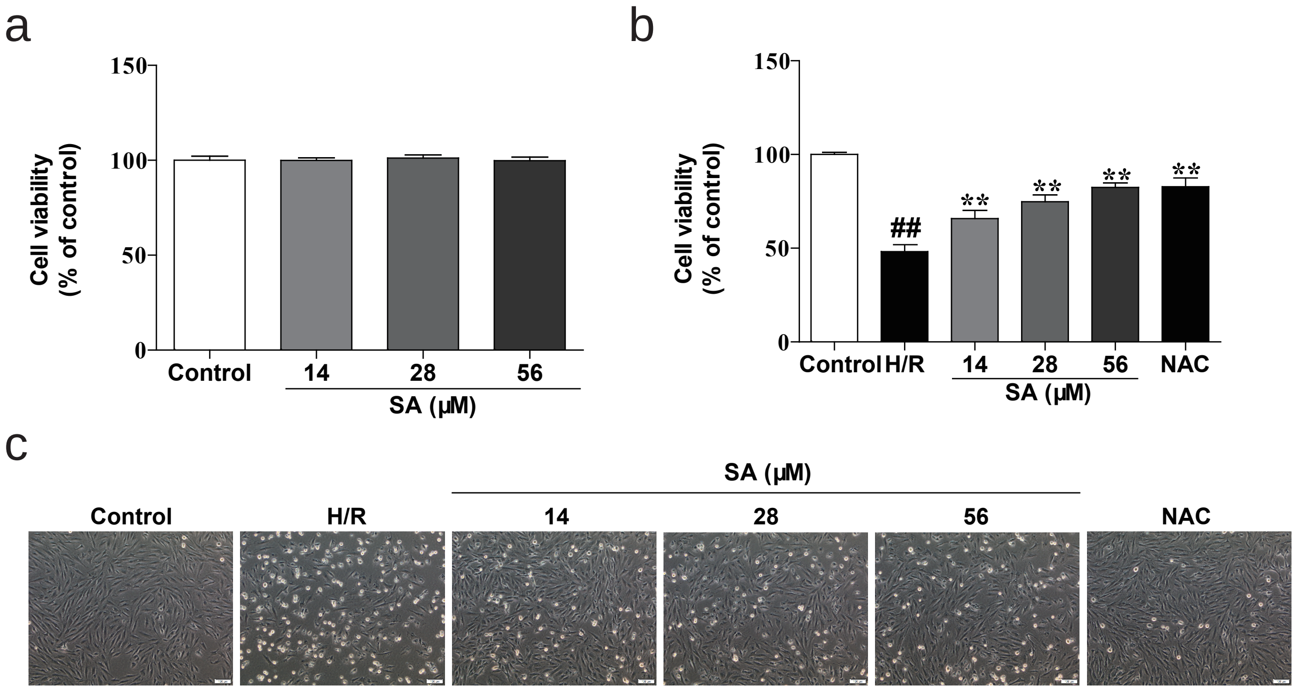

Figure S1: SA protected cardiomyocytes against injury induced by H/R. H9c2 cells were treated with SA and then exposed to hypoxia of 6 h followed by 6 h reoxygenation. (a) Effect of SA on H9c2 cardiomyocytes. (b) Cell viability. (c) Representative images of cell morphology. The data were expressed as mean ± SEM. $^{##}P<0.01$ vs. Control group; $^{**}P<0.01$ vs. H/R group.

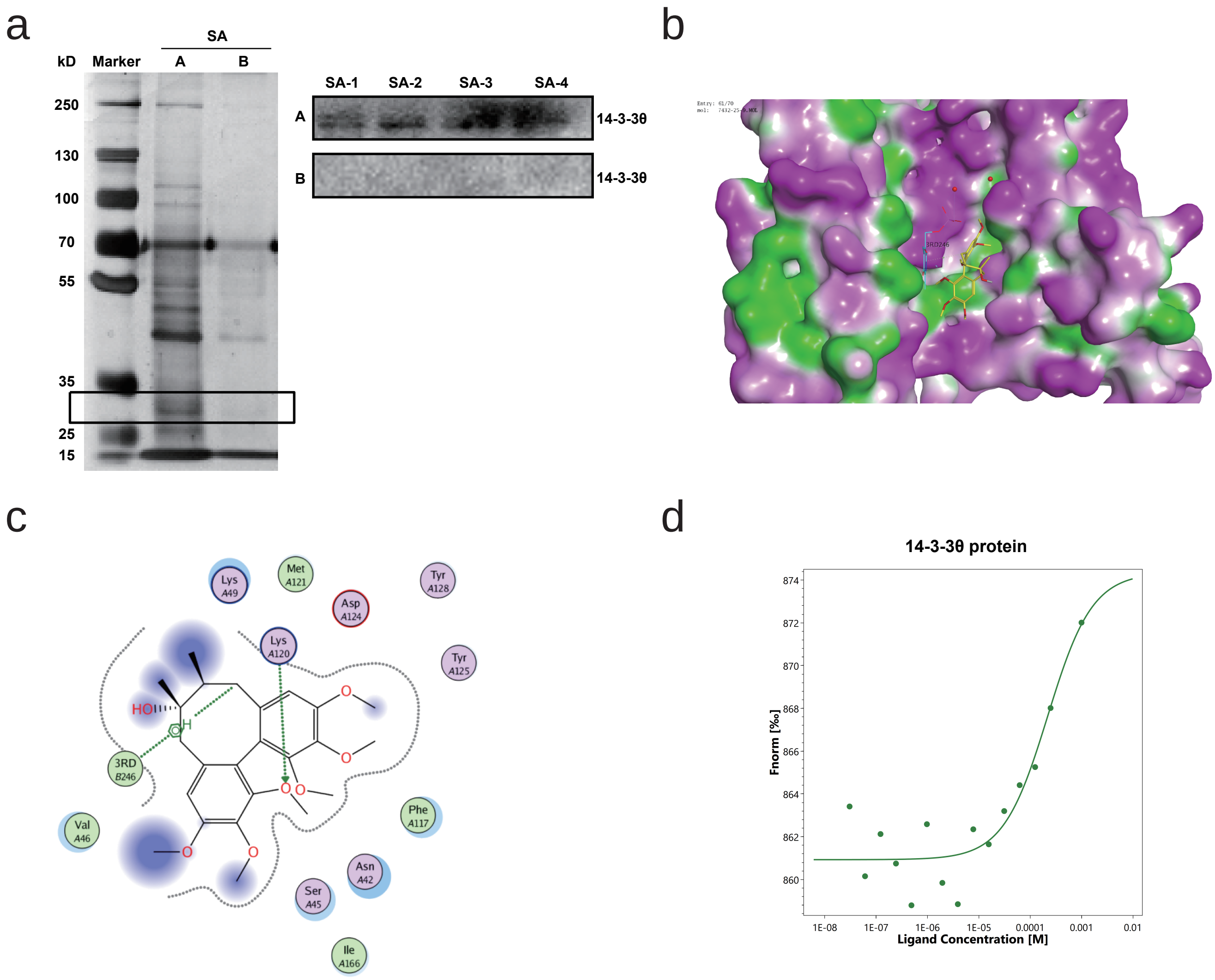

Figure S2: Target screening and validation of SA. (a) Capture and identification of target proteins using serial affinity chromatography. (b) Molecular docking of 14-3-3θ and SA. (c) Software analysis was performed to screen out possible amino acids for interaction. (d) The combining capability between SA and 14-3-3θ protein was detected by MST analysis.
